# Chromatin Reorganization during Myoblast Differentiation Involves the Caspase-Dependent Removal of SATB2

**DOI:** 10.3390/cells11060966

**Published:** 2022-03-11

**Authors:** Ryan A. V. Bell, Mohammad H. Al-Khalaf, Steve Brunette, Dalal Alsowaida, Alphonse Chu, Hina Bandukwala, Georg Dechant, Galina Apostolova, F. Jeffrey Dilworth, Lynn A. Megeney

**Affiliations:** 1 Regenerative Medicine Program, Sprott Center for Stem Cell Research, Ottawa Hospital Research Institute, The Ottawa Hospital, Ottawa, ON K1H 8L6, Canada; rybell@ohri.ca (R.A.V.B.); mal-khalaf@ottawaheart.ca (M.H.A.-K.); sbrunette@ohri.ca (S.B.); dalsowaida@ohri.ca (D.A.); alphonse.chu@yahoo.ca (A.C.); hbandukwala@ohri.ca (H.B.); jdilworth@ohri.ca (F.J.D.); 2 Department of Cellular and Molecular Medicine, University of Ottawa, Ottawa, ON K1H 8M5, Canada; 3 University of Ottawa Heart Institute, Ottawa, ON K1Y 4W7, Canada; 4 Institute of Neuroscience, Medical University of Innsbruck, A-6020 Innsbruck, Austria; georg.dechant@i-med.ac.at (G.D.); galina.apostolova@i-med.ac.at (G.A.)

**Keywords:** SATB2, chromatin remodeling, caspase 7, myogenic differentiation

## Abstract

The induction of lineage-specific gene programs are strongly influenced by alterations in local chromatin architecture. However, key players that impact this genome reorganization remain largely unknown. Here, we report that the removal of the special AT-rich binding protein 2 (SATB2), a nuclear protein known to bind matrix attachment regions, is a key event in initiating myogenic differentiation. The deletion of myoblast SATB2 in vitro initiates chromatin remodeling and accelerates differentiation, which is dependent on the caspase 7-mediated cleavage of SATB2. A genome-wide analysis indicates that SATB2 binding within chromatin loops and near anchor points influences both loop and sub-TAD domain formation. Consequently, the chromatin changes that occur with the removal of SATB2 lead to the derepression of differentiation-inducing factors while also limiting the expression of genes that inhibit this cell fate change. Taken together, this study demonstrates that the temporal control of the SATB2 protein is critical in shaping the chromatin environment and coordinating the myogenic differentiation program.

## 1. Introduction

One of the key alterations that characterize a cell’s progression through differentiation is the restructuring of the nuclear landscape to allow for the expression of lineage-specific genes. Indeed, chromatin conformation undergoes significant restructuring when cells progress from replicating to differentiating phenotypes, with changes occurring to repress or open access at specific gene loci [1,2]. These genome alterations are facilitated by two general mechanisms—loci-specific modifications of DNA and histones and the targeting of structural proteins that control the higher-order structure of chromatin [3,4]. Progress through the myogenic differentiation program is no exception to this paradigm, with extensive changes to genome structure during myogenesis that allow for muscle-specific genes to be expressed [5]. A number of proteins that govern DNA and histone methylation changes have been implicated in the control of myogenesis [6], yet limited information exists on the role of proteins that manage higher-order reorganization such as the formation of chromatin loops and the delineation of topologically associating domains (TADs).

Matrix attachment region (MAR) binding proteins represent a group of factors proposed to act as modifiers of the chromatin loop structure to influence gene expression during cell differentiation. MAR binding proteins are unique in that they integrate both repressive and inductive signals for gene expression, a feature that would be valuable for the management of cell fate transitions. This duality of MAR protein function depends on the proximity of gene promoters and insulator regions relative to the position where the MAR protein anchors the DNA to the chromatin scaffold [7,8]. Scaffold attachment factor b1 (Safb1) has been shown to activate pro-differentiation gene expression in skeletal muscle cells, yet it does not appear to directly repress the expression of genes in muscles [9].

The special AT-rich binding proteins (SATB1 and SATB2) are a family of MAR binding proteins that may play an active role in global chromatin organization during cell differentiation. Although SATB proteins were originally defined as classic chromatin structure factors, a number of studies have suggested their direct influence on gene expression, akin to the biology of a transcription factor. For example, SATB2 has been found to control the expression of several genes involved in stem cell fate determination and cancer cell proliferation, acting in concert through physical association with other transcription factors [10,11,12,13,14,15,16]. However, one recent observation has shown that SATB2 can modify gene expression in cortical neurons through its association with the inner nuclear membrane protein, LEMD2 [17]. This latter study is more consistent with the structural role of SATB2, and the plethora of developmental abnormalities that accompany SATB2 mutation in humans suggest a genome-spanning function for this MAR protein [18]. Given these observations, the regulation of the SATB2 protein content may be a critical step in managing the duality of gene expression during cell maturation, i.e., the simultaneous repression and activation of targeted gene loci.

The mechanism by which SATB2 manages such a diverse biologic response is unknown. Likewise, there are no studies that have characterized the SATB2-dependent genome-wide architecture or the mode by which the SATB2 protein is targeted or displaced from its associated genomic targets. However, the caspase-mediated cleavage of SATB1 has been shown to be an important step in the control of gene expression that precedes T-cell apoptosis, where the cleavage of SATB1 results in its release from MARs [19]. Interestingly, caspase proteases have prominent roles in non-death processes such as differentiation, inflammation, remodeling, and cell survival [20]. The role of caspases in differentiation is particularly well-established, with both initiator and executioner caspases being involved in the development of a variety of tissues [21]. With respect to skeletal muscle differentiation, catalytically active caspase 3 and 9 appear to be required for progenitor progression through the myogenic program [22,23,24]. As such, the integral nature of caspase activity during myoblast differentiation and the possibility that caspases could act on chromatin organizing proteins have led us to investigate the behavior of SATB2 within muscle progenitor cells and the potential of caspase enzymes to mediate its function.

## 2. Materials and Methods

### 2.1. Mice and In Vivo Procedures

All mice were housed and treated at the University of Ottawa Animal Care and Veterinary Services. Mice used in our studies were housed and cared for according to Canadian Council on Animal Care (CCAC) guidelines and University of Ottawa Animal Care Committee protocols.

In order to determine the effects of SATB2 removal from muscle satellite cells, *Pax7*/*CreER Satb2^fl^*^/*fl*^ and *Satb2^fl^*^/*fl*^ (control) mice were fed a diet supplemented with tamoxifen (40 mg/kg body weight; Envigo) at 3 weeks old. These mice were monitored weekly for evidence of the potential effects of SATB2 ablation from muscle stem cells. After 8–10 months, three *Pax7*/*CreER Satb2^fl^*^/*fl*^ and *Satb2^fl^*^/*fl*^ mice (female) were euthanized by CO_2_ asphyxiation and cervical dislocation. These mice were then assessed for any structural abnormalities, and their tibialis anterior (TA) muscle was excised and fixed in 10% neutral buffered formalin. After 24 h, the samples were then placed in 70% ethanol and then paraffin embedded and cut into 4 μm sections.

### 2.2. Cell Culture

Cells of the immortalized mouse cell line, C2C12, were grown on non-collagen coated cell culture plates in Dulbecco’s Modified Eagle’s medium (DMEM) with 10% fetal bovine serum (FBS) and 1% penicillin/streptomycin (growth medium). Once confluent, the cells were differentiated in DMEM supplemented with 2% horse serum and 1% penicillin/streptomycin (differentiation medium).

Primary myoblasts were isolated, as described by Fernando et al. [22]. Following isolation, myoblasts were cultured in 1:1 (*v*/*v*) of Ham’s F10:DMEM, supplemented with 20% FBS, 1% penicillin/streptomycin, and 10 ng/mL basic fibroblast growth factor. Myoblasts were differentiated in DMEM supplemented with 5% horse serum and 1% penicillin/streptomycin.

### 2.3. Protein Extraction and Western Blotting

C2C12 cells were lysed using a modified RIPA buffer (50 mM Tris-HCl, pH 7.4, 150 mM NaCl, 1 mM ethylenediaminetetraacetic acid (EDTA), 1% NP-40, 1% glycerol, and a cocktail of protease inhibitors) for whole-cell extracts, or with a modified NE-PER Nuclear and Cytoplasmic Extraction Kit, as per the manufacturer’s instructions (Thermo Scientific, Waltham, MA, USA). Extracted proteins were then separated via SDS-PAGE and then transferred to a 0.45 μM polyvinylidene fluoride (PVDF) membrane (Millipore, Burlington, MA, USA) on a TRANS-BLOT SD apparatus (Bio-Rad, Hercules, CA, USA). Membranes were blocked with Tris-buffered saline plus 0.1% Tween-20 (TBST) containing 5% skim milk for 1 h at room temperature. Membranes were then incubated overnight at 4 °C in primary antibody made with the blocking solution. The primary antibodies used in this study included mouse SATB2 (ab51502; Abcam, Cambridge, UK; 1:1000), rabbit cleaved caspase 7 (9491S; Cell Signaling, Danvers, MA, USA; 1:1000), mouse myosin heavy chain (Development Studies Hybridoma Bank, DSHB Iowa City, IA, USA; MHC; 1:250), rabbit lamin A/C (2032; Cell Signaling; 1:1000), mouse tubulin (DSHB; 1:200), and mouse glyceraldehyde 3-phosphate dehydrogenase (#2118; GAPDH; 1:4000). Western blots were performed on three independent cell preparations in all cases (*n* = 3). 

### 2.4. Immunofluorescence

C2C12 cells or primary myoblasts were cultured and fixed in paraformaldehyde on 25 mm coverslips at the desired time points. The cells were then incubated for 10 min at room temperature with a permeabilization solution containing 0.5% Triton-X 100 in PBS. Subsequently, after washing in PBS, cells were incubated for 1 h in a blocking solution consisting of 5% horse serum in PBS. After blocking, the cells were incubated for 2 h at room temperature or overnight at 4 °C in primary antibody that was diluted in blocking solution. The primary antibodies used were mouse SATB2 (1:50; ab51502, Abcam), rabbit heterochromatin protein 1 alpha (HP1α; 1:200; #2616, Cell Signaling), rabbit GAPDH (1:400; #2118, Cell Signaling), rabbit desmin (1:400; ab15200, Abcam), and mouse MHC (1:50; DSHB). After primary antibody incubation, the cells were washed three times in PBS and incubated in secondary antibody (2 mg/mL Alexa Fluor 488, Invitrogen, Waltham, MA, USA; 1:1500, 2 mg/mL Alexa Fluor 594, Invitrogen, 1:1500; 2 mg/mL Alexa Fluor 568, Invitrogen, 1:1000) diluted in PBS for 1.5 h at room temperature. After incubation, the cells were washed 2× in PBS and then counterstained with 4′,6-diamidino-2-phenylindole dihydrochloride (DAPI; 1:10,000; Sigma) for 10 min at room temperature. After incubation, the cells were washed 2× in PBS, and the coverslips were mounted on microscope slides using Dako Fluorescent Mounting Medium. The cells were then visualized using a Zeiss Observer Z1 inverted fluorescence microscope, Zeiss Canada, Toronto, ON, Canada. All images were captured using the AxioVision 4.8 software. Quantification of immunofluorescence was performed by densitometry analysis using the ImageJ/Photoshop C3 software (Public Domain). Immunofluorescence experiments were performed on *n* = 3–5 independent cell preparations depending on the experiment (detailed in the figure captions).

### 2.5. Immunohistochemistry

Mouse TA muscle was fixed in 10% formalin for 24–48 h before being embedded in paraffin and sectioned into 4 µm thick sections. The sections were then stained for Pax7 (DSHB hybridoma antibody; 1:3.5), SATB2 (Abcam; 1:300), or with hematoxylin and eosin (H&E). The control for IHC staining was staining that used just secondary antibody that originated from the same species as the primary antibody used. The sectioning and staining were conducted by the Pathology and Laboratory Medicine (PALM) Histology Core Facility at the University of Ottawa, Canada. To determine muscle fiber areas, H&E stained sections were assessed under an Observer A1 microscope (Zeiss), and areas were calculated using the ImageJ software.

### 2.6. Chromatin Immunoprecipitation (ChIP) Assay

C2C12 cells were grown on 15 cm plates and allowed to reach 100% confluence before three aliquots of twenty million proliferating cells were collected (technical replicate *n* = 3; biological replicate *n* = 1). Cells were fixed for 10 min using 1% formaldehyde in DMEM. Cell fixation was quenched by removing the fixation solution, rinsing the plates with PBS, and then pouring a solution of 0.125 M glycine in PBS onto the cells and incubating them for 5 min. Subsequently, the plates were washed 2× with PBS, and then cells were scraped from the plates, pelleted, and stored at −80 °C until used.

Given that SATB2 is a nuclear matrix attachment protein, it required an alternative protocol for chromatin shearing as compared to standard preparations. First, a commercial hypotonic solution (Active Motif, Carlsbad, CA, USA) was used to lyse cellular membranes but retain intact the nuclear membranes. We subsequently performed additional lysis using a handheld glass Dounce homogenizer. After centrifugation, the supernatant was discarded, and the pelleted nuclei were treated with Active Motif’s Pro-enzymatic Digestion Nuclear Extraction Solution. After incubation at 37 °C for 5 min, we supplemented the reaction with 1 U micrococcal nuclease (New England Biolabs Canada, Toronto, ON, Canada) and allowed it to incubate for 10 min. We then supplemented the reaction with SDS to a final volume of 2% SDS-chromatin solution. We proceeded to the second stage of shearing using sonication via a Covaris M220 Focused-Ultrasonicator instrument, setting the parameters to produce sheared DNA of 200 bp. After sonication, we performed a final centrifugation at 16,000× *g* at 4 °C for 20 min and collected the supernatant containing the sheared chromatin. 

We performed immunoprecipitations with 5 μg 1°Ab (Target: SATB2, Abcam; positive control: RNA pol II, Active Motif; negative control: mouse IgG, Santa Cruz, Dallas, TX, USA) using magnetic beads (Active Motif) diluted in a commercial ChIP-buffer (Active Motif) overnight at 4 °C. Captured chromatin was isolated using magnetic stands to pull down the beads. Bound DNA was subsequently eluted via commercial elution, reverse cross-linking, protein digestion, and RNA digestion solutions (Active Motif). The resulting samples of DNA were further purified using phenol:chloroform extraction procedures. Final DNA was quantified using a NanoDrop spectrophotometer, with a 25 μL final volume at concentrations of 75–100 ng/μL.

### 2.7. ChIP-Sequencing and Bioinformatics

For the genome-wide analysis, immunoprecipitated DNA was amplified and a 75 bp single-read sequencing was performed on an Illumina HiSeq 2500 at the Next-Generation Sequencing Facility at The Centre for Applied Genomics in The Hospital for Sick Children (Toronto, ON, Canada). The data were summarized, and basic comparisons were performed using the Excel spreadsheet program (Microsoft, Redmond, WASH, USA). Reads were aligned to the GRCm38 (UCSC mm10, Dec/2011) mouse genome from the UCSC genome browser with bowtie2 (v2.2.6) using the default parameters, and peaks were called using MACS version 2.1.0, again with default parameters and using the IgG ChIP-seq data as a control (Supplementary Table S4). 

The peaks identified by MACS were analyzed in R using the ChIPseeker package [25] to identify sites of SATB2 enrichment relative to genes (promoter, exon, intron, UTR, etc.). Genes with one or more SATB2 binding sites in a region from −5 kb to + 1 kb around the transcription start site (TSS) were identified as being associated with Satb2 binding. This gene list was analyzed with ClusterProfiler [26] to identify Gene Ontology (GO) terms for which genes bound by SATB2 are statistically enriched above the expected level using the hypergeometric test. HOMER v4.11.1 [27] was used to search for enriched DNA sequence motifs within the full set of Satb2 peaks, using the default parameters for the findMotifsGenome.pl tool.

### 2.8. RNA-Sequencing and Bioinformatics

Total RNA was isolated with an RNeasy Kit (QIAGEN, Hilden, Germany) using an on-column DNase digestion (RNase-Free DNase Set, QIAGEN) to avoid genomic DNA contamination. Library preparation and 126-bp paired-end RNA-seq was performed by the Next-Generation Sequencing Facility at The Centre for Applied Genomics in The Hospital for Sick Children. RNA integrity was assessed using the Bioanalyzer platform (Agilent Technologies, Inc. Santa Clara, CA, USA). Sequencing was performed using standard procedures for the Illumina HiSeq 2500 platform. 

RNA-seq reads were mapped to the GRCm38 (mm10) mouse genome assembly using tophat2 v2.1.1 [28], and the mapped reads were assigned to transcripts from the GENCODE vM23 annotation using FeatureCounts v1.5.2 [29]. Read count data for 24 h differentiated siControl and siSATB2 C2C12 cells, with three replicates each, were loaded into R (v4.0.2), and the differential expression was assessed using DESeq2 v1.30.1 [30]; expression differences between the siSatb2 and siControl replicates were calculated using the DESeq2 lfcShrink function and applying the apeglm method (v 1.12.0. [31]). Multiple testing correction was performed using the Benjamini Hochberg method, and lists of significantly differentially expressed (DE) genes were identified using a q-value (i.e., a corrected *p* value) cut-off of 0.05.

The matrix of gene expression values was transformed with the DESeq2 rlog function, and the DESeq2 plotPCA function was used to run a principal component analysis of the 500 most variable genes and to plot the results of the first two principal components. Sets of genes that were significantly higher or lower in abundance in siSatb2 cells (2013 and 1914 genes, respectively) were analyzed using the clusterProfiler package to identify the GO terms for which genes in either list were significantly over-represented using a hypergeometric test. Gene lists were also uploaded to the gProfiler tool [32] to identify enriched pathways in GO datasets.

GSEA was performed using the R package fgsea v1.14.0. In brief, a ranking statistic was first derived for all differentially expressed genes identified by DESeq2. This was performed by multiplying the sign of the Log2FoldChange with the respective log10 transformed *p* value for each gene. After arranging the genes using the signed *p* value statistic, GSEA was performed with the ranked gene list and the REACTOME Canonical Pathway v7.5 from the Molecular Signature Database (MSigDb). Parameters of note that were used with the fgsea function include eps = 0.0, minSize = 15, and maxSize = 500 [33].

### 2.9. Chromosome Conformation Capture, Hi-C

siControl and siSatb2-treated C2C12 cells were prepared for Hi-C experiments, as described below in the section illustrating the siRNA-mediated knockdown of SATB2. Only proliferating C2C12 cells were assessed via Hi-C. Once the cell treatments were complete, C2C12s were counted using trypan blue and a hemocytometer so that each sample would have 1 × 10^7^ cells/sample. Two independent samples were collected for each treatment. Each aliquot of cells was pelleted at 500× *g* for 5 min and then resuspended in 10 mL PBS. Cells were then fixed in 8% formaldehyde for 10 min, after which the cross-linking reaction was quenched in 0.65 M glycine for 5 min. This mixture was allowed to sit in wet ice for 15 min before the cells were pelleted at 500× *g* for 5 min. C2C12s were subsequently resuspended in 5 mL PBS and then pelleted once again at 500× *g* for 5 min. The supernatant was then aspirated, and the pellet was frozen on dry ice for 10 min before being stored at –80 °C. The samples were then shipped on dry ice to Arima Genomics (http://arimagenomics.com (accessed on 30 April 2021) San Diego, CA, USA), which performed the Hi-C analysis according to the Arima-HiC + protocols described in the Arima-HiC kit (P/N: A510008). After the Arima-HiC protocol, Illumina-compatible sequencing libraries were prepared by first shearing the purified Arima-HiC proximally ligated DNA and then size-selecting the DNA fragments of ~200–600 bp using SPRI beads. The size-selected fragments were then enriched for biotin and converted into Illumina-compatible sequencing libraries using the KAPA Hyper Prep kit (P/N: KK8504). After adapter ligation, the DNA was PCR amplified and purified using SPRI beads. The purified DNA underwent standard quality control (qPCR and Bioanalyzer) and was sequenced on the HiSeq X, following the manufacturer’s protocols.

### 2.10. Hi-C Data Processing

Hi-C data were analyzed with Juicer v1.6 [34] using a custom restriction site file for the GRCm38 mouse genome assembly provided by Arima Genomics (GATC and GANTC sites). Data from the replicates (*n* = 2) were combined for the siControl and siSatb2-treated samples and subsequently used for any downstream analysis. Chromatin loops were identified using HiCCUPS from Juicer v1.6 with the default settings. Loops were delineated at resolutions of 25,000, 10,000, and 5000 bp and thereafter merged to generate a final loop set. HiCexplorer v3.6 was used to identify topologically associated domains (TADs; 100 kb, 500 kb, and 1 Mb resolution) and sub-TAD contact domains (10 kb resolution). With respect to TADs, only data from the 100 kb resolution was depicted. The function *hicFindTADs* was used with the Knight–Ruiz (KR) normalized Hi-C matrices with a *p* value cut-off of 0.05 and FDR 0.01. ChIPseeker v1.24.0 was used for the functional annotation of the domains. 

SATB2 binding sites and chromatin loops/TADs were compared to repressive (H3K27me3) and permissive (H3K4me3 and H3K36me3) histone marks that had been generated by Asp et al. [35] from proliferating C2C12 myoblasts. The accession numbers for the associated ChIP-seq data are: H3K4me3 (GEO accession: GSM721292), H3K27me3 (GEO accession: GSM721294), and H3K36me3 (GEO accession: GSM721296). Single-end reads were downloaded from the GEO database and mapped to mm10 using Bowtie2 v2.4.2. After removing duplicates with Picard Tools (openjdk v1.8.0_292) and MAPQ-based filtering (-q 10) with Samtools v1.12, broadPeaks were called using Macs3 v3.0.0a6 (–broad-cutoff 0.05 -*p* 0.05 –keep-dup 1).

Similarly, SATB2 ChIP-seq peaks and chromatin structural features determined here by Hi-C were compared to known CTCF binding sites. Processed data for CTCF ChIP-seq data in mouse C2C12 cells were obtained directly from ENCODE (ENCSR000AIJ). The processed data included: (1) reads aligned to mm10 in BAM format (ENCSR000AIJ), and (2) IDR thresholded peaks in bed narrowPeak format (ENCFF784ASD).

### 2.11. Caspase Cleavage Assays

Recombinant SATB2 protein (250–500 ng; Abnova) and recombinant active caspase 3 (0.5 μg; Chemicon, Thermo Fisher Scientific, Waltham, MA, USA) or recombinant active caspase 7 (0.5 μg; BioVision, Milpitas, CA, USA) were incubated for 3 h in a cleavage assay buffer (50 mM Hepes, pH 7.5, 0.1 M NaCl, 10% (*v*/*v*) glycerol, 0.1% Chaps, 10 mM dithiothreitol) containing either dimethyl sulphoxide (DMSO) or z.DEVD.fmk (20 μM; BioVision; [36,37]), as indicated. Reactions were stopped by the addition of the Laemmli sample buffer and subjected to SDS-PAGE. Mass spectrometry was performed at the Ottawa Hospital Research Institute Proteomics Core Facility (Ottawa, Canada). MASCOT 2.3.01 software (Matrix Science, Boston, MA, USA) was used to infer peptide and protein identities from the mass spectra.

### 2.12. Caspase Inhibition Assays

For caspase 3/7 inhibition, cultured C2C12s were pre-treated with either 15 μM z-DEVD-fmk (DEVD) from BioVision or 15 μM DMSO from Sigma for 2 h at 37 °C. After pre-treatment, the cells were induced to differentiate using low serum media or continued in growth media both containing 15 μM DEVD or DMSO as a vehicle-only control. The inhibition or control media were changed every 48 h until the end of the time course. Cells were collected at the predetermined time points and analyzed as described. Caspase inhibition assays were performed on *n* = 3 independent cell preparations.

### 2.13. siRNA-Mediated Depletion of SATB2 and Caspase 7 Gene Expression

siRNA duplexes were used to suppress *Satb2* and caspase 7 gene expression in C2C12 cells. C2C12 cells were transfected at 25% confluence with 10 nM siRNA (siSatb2, siCasp7, or siControl) and the Lipofectamine RNAiMAX reagent, as directed by the manufacturer’s protocol (Invitrogen). After an overnight incubation, fresh media were added onto the cells, and the cells were re-transfected. This continued until the cells reached 100% confluence, after which differentiation media were added to the cells. Media change and re-transfection occurred every 24 h throughout the indicated time course.

### 2.14. Statistical Analysis

Statistical analysis of three or more data sets was performed using one-way analysis of variance (ANOVA), with the post hoc test being indicated in the figure caption. For comparison between two sample sets, an unpaired, two-tailed Student’s *t*-test was performed, unless otherwise stated, and a *p* < 0.05 was considered statistically significant. Data represented by bar graphs are all mean ± standard error of the mean (SEM).

## 3. Results

### 3.1. SATB2 Restrains the Induction of Muscle Cell Differentiation 

**Figure 1:**
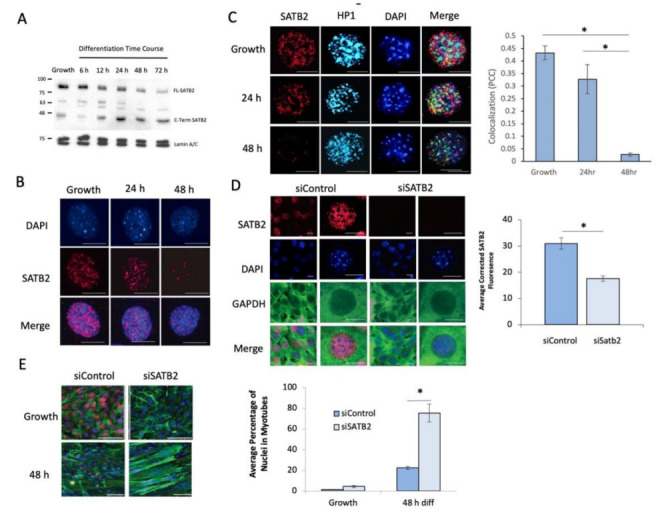
Loss of SATB2 expression accelerates myoblast differentiation. (**A**) Representative western blot showing the decreased expression of full-length SATB2 (FL-SATB2) during a differentiation time course for C2C12 cells (*n* = 3). This was accompanied by a concomitant increase in a C-terminal fragment of SATB2 (C-Term SATB2; identified in Supplementary Table S1). (**B**) Representative immunofluorescent staining of SATB2 showing a decrease in SATB2 expression during early C2C12 cell differentiation. Images are representative of *n* = 5 independent samples at each time point. DAPI (blue), SATB2 (red); scale = 10 μm. (**C**) Representative immunofluorescent images depicting SATB2 localization in relation to heterochromatin protein 1α (HP1α). Images are representative of *n* = 3 independent determinations for each time point. Scale bar = 10 μm. DAPI (blue), SATB2 (red), HP1α (teal). Bar graph depicts colocalization of SATB2 and HP1 within the nucleus of proliferating and differentiating myoblasts. Data are the quantification of immunofluorescent images to the left of the graph. * indicates a significant change in colocalization, determined using a one-way ANOVA with a post hoc Tukey test (*p* < 0.01). Data are means ± SEM for *n* = 3 independent determinations. (**D**) Immunofluorescent images depicting the efficacy of SATB2 depletion using siRNA in proliferating C2C12 cells. Muscle cell cytoplasm was counterstained with an anti-GAPDH antibody. Images are representative of *n* = 3 independent determinations. DAPI (blue), GAPDH (green), and SATB2 (red). Scale bar for the low magnification column = 10 μm. Scale bar for the higher magnification column = 5 μm. Bar graph depicts the quantification of SATB2 expression from the immunofluorescent images. * indicates a significant difference in SATB2 expression between siControl and siSATB2 conditions using the Student’s *t*-test (*p* < 0.05). Data are means ± SEM for *n* = 3 independent determinations. (**E**) Immunofluorescent images indicating that the depletion of SATB2 led to early myotube formation after C2C12 cells were induced to differentiate. Images are representative of *n* = 3 independent determinations. DAPI (blue), SATB2 (red), and desmin (green). Scale bar = 50 μm. Bar graph depicts the fusion index for proliferating (Growth) and 48 h differentiated myoblasts. Data were a consequence of assessing immunofluorescent images depicted in Figure 1E. * indicates a significant change in the fusion index following siSATB2 treatment as compared to siControl-treated cells, determined by the Student’s *t*-test (*p* < 0.05). Data are means ± SEM for *n* = 3 independent determinations.

Western blot and immunofluorescent analyses identified SATB2 as a nuclear protein within proliferating C2C12 muscle cells, which was markedly reduced in expression during the early stages of differentiation (Figure 1A,B and Figure S1A). This signal was not confounded by potential cross reactivity with the closely related protein, SATB1, as this protein was not detected in proliferating or differentiated myoblasts (Supplementary Figure S1F). When expressed, SATB2 was mainly relegated to the euchromatic nuclear space; however, there was a significant subfraction of SATB2 co-staining with HP1α occurring in the more condensed regions of the nucleus, which declined as the differentiation program proceeded (Figure 1C). Having established that the SATB2 protein is reduced during myogenesis, we next sought to determine the role of SATB2 in muscle cell proliferation and differentiation. To this end, we initially designed CRISPR-Cas9 guide RNAs to inhibit SATB2 gene expression in replicating myoblasts. However, infection of C2C12 muscle cells with an adenovirus expressing enhanced Cas9 [38] led to a notable increase in SATB2 protein content in post-differentiated myoblasts, at a time when SATB2 would otherwise be in significant decline (see Supplementary Figure S1D). Given this unexpected impact of Cas9 on our protein of interest, we chose to pursue the siRNA-mediated targeting of SATB2 as a means of addressing its biologic role in cell culture models. The siRNA targeting of SATB2 (siSATB2) did not produce any noticeable alteration in cell viability or growth (Supplementary Figure S1B,C), although it provided an effective decrease in SATB2 expression (Figure 1D). However, compared to wildtype cells, siSATB2-treated cells displayed altered differentiation kinetics, with the accelerated formation of multinucleated myotubes (Figure 1E) and the increased expression of the contractile protein, myosin heavy chain (MHC) (Supplementary Figure S1E). 

### 3.2. Caspase 7 Cleaves SATB2 during Early Myogenesis

**Figure 2:**
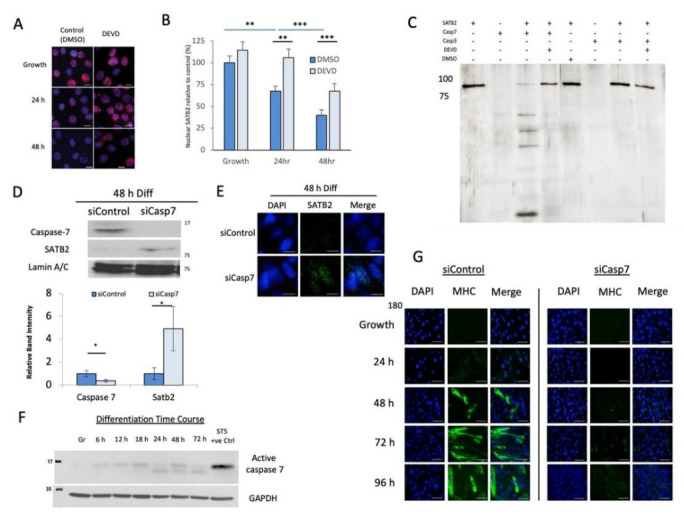
Caspase 7 cleaves SATB2 during early skeletal muscle differentiation and is necessary for myogenesis. (**A**) Immunofluorescent staining showing the maintenance of SATB2 expression following caspase inhibition by z-DEVD.fmk (DEVD) in differentiating C2C12 cells. Images are representative of *n* = 3 determinations on independent samples. DAPI (blue); SATB2 (red); scale: 10 μm. (**B**) Quantification of SATB2 nuclear expression following DEVD-mediated suppression of caspase activity. Data are the means ± SEM from *n* = 3 independent samples; 75–100 cells per condition/time point were analyzed. ** indicates *p* < 0.01 and *** indicates *p* < 0.001, as determined by the Student’s *t*-test. (**C**) Silver stained gel of an in vitro caspase cleavage assay showing SATB2 cleavage by caspase 7. Reactions include combinations of recombinant SATB2 protein, active caspase 3/7 recombinant proteins, caspase chemical inhibitor (DEVD), or the control chemical DMSO (*n* = 3). (**D**) Caspase 7 and SATB2 expression following siCasp7 and siControl treatments of C2C12 cells. Both the histogram and the representative western blots (*n* = 3) were the expression levels of those two proteins 48 h post-induction of differentiation. * indicates a significant difference in caspase 7 or SATB2 expression, as determined by the Student’s *t*-test (*p* < 0.05). (**E**) Immunofluorescent images showing the sustained expression of SATB2 after the depletion of caspase 7 in C2C12 cells differentiated for 48 h. Images are representative of *n* = 3 independent determinations. Scale bars = 10 μm. (**F**) Representative western blot depicting the cleaved active fragment of caspase 7 found in the nuclear fraction of C2C12 cells during their proliferative phase (Gr) and at various stages of differentiation (6–72 h post-induction; *n* = 3). Staurosporine (STS) was administered to proliferating C2C12 cells to induce apoptosis as a positive control. (**G**) Immunofluorescent images showing the reduction in myosin heavy chain (MHC) expression in differentiating C2C12 myoblasts. Panel is representative of *n* = 3 determinations on independent samples. DAPI (blue), MHC (green). Scale bars = 50 μm.

Evidence from apoptotic nuclei suggests that nuclear structural proteins are targeted by caspase proteases [19,39,40]. Moreover, our identification of a major C-terminal fragment of SATB2 that becomes more prominent during muscle cell differentiation suggests that a proteolytic event may target the SATB2 protein (Figure 1A and Figure S2D). Prior studies from our laboratory have shown that caspase 3 plays a prominent role in myogenesis, targeting and cleaving a number of substrate proteins to engage the differentiation program [22,24,36]. To examine whether SATB2 proteolysis occurred, we suppressed endogenous effector caspase activity (caspase 3 and 7) with the peptide inhibitor z.DEVD.fmk and reassessed SATB2 protein expression during muscle cell differentiation. Caspase inhibition during differentiation led to the sustained expression of the SATB2 protein in the nucleus compared to control cells (Figure 2A,B). In an attempt to attribute SATB2 cleavage to either caspase 3 or 7 (or both), an in vitro cleavage assay was performed. This assay demonstrated that caspase 7 activity was very robust at targeting SATB2 protein, whereas caspase 3 did not induce measurable cleavage (Figure 2C). A mass spectrometry analysis of the caspase 7-mediated SATB2 fragments mapped a putative caspase cleavage site at D477 (Supplementary Figure S2C and Table S1). 

To corroborate caspase 7 as a targeting protease of SATB2 and by inference as a regulatory enzyme that may promote muscle cell differentiation, we utilized siRNA to suppress caspase 7 expression. siRNA repression of caspase 7 (siCasp7) led to a concomitant accumulation of the SATB2 protein during C2C12 muscle cell differentiation when compared to siControl-treated cells (Figure 2D,E and Figure S2A). Investigating caspase 7 more closely, we determined that the expression of nuclear, active caspase 7 was inversely correlated with SATB2 expression, with nuclear caspase 7 levels increasing during early myoblast differentiation (Figure 2F and Figure S2E). To assess the broad effect of this protease on muscle differentiation, we compared siCasp7 cells (where siRNA treatment reduced caspase 7 expression by ~60%; Figure 2D) vs. siControl-treated cells and noted that siCasp7 cultures displayed a significant impairment in the low serum induction of differentiation, with a dramatic reduction in the expression of MHC and a near complete inhibition of multi-nucleate myotube formation (Figure 2G). Interestingly, the simultaneous suppression of caspase 7 and SATB2 via RNA interference caused a modest increase in MHC expression from levels observed when only caspase 7 was repressed (Supplementary Figure S2B).

### 3.3. ChIP- and RNA-seq Data Support the Role of SATB2 in Regulating Muscle Satellite Cell Differentiation and Chromatin Reorganization

**Figure 3:**
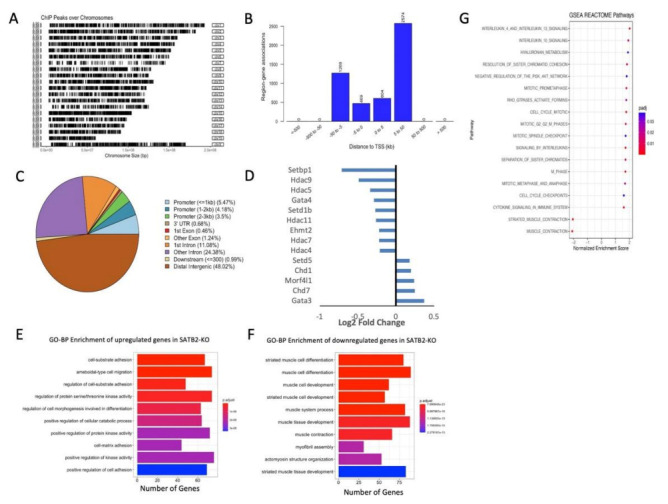
SATB2 regulates the expression of genes associated with chromatin remodeling and cell differentiation. (**A**) Chromosome distribution of SATB2 binding sites as determined from our ChIP-seq analyses (using ChIPseeker) on proliferating C2C12 cells. (**B**) Distribution of SATB2 binding sites within the muscle satellite cell genome with respect to the closest associated gene transcription start site (TSS). (**C**) Genomic context of SATB2 binding sites as determined by ChIPseeker. (**D**) Differentially expressed chromatin remodeling genes after inhibition of *Satb2* expression in 24 h differentiated myoblasts, as determined using DESeq2 following our RNA-seq experiments. (**E**) Top GO biological process (BP) categorization of the genes upregulated following *Satb2* repression in 24 h differentiated myoblasts. (**F**) Top GO-BP categorization of the downregulated genes following *Satb2* siRNA-mediated repression in 24 h differentiated myoblasts. Analyses in (**E**,**F**) were provided by ClusterProfiler. (**G**) Gene Set Enrichment Analysis (GSEA) for differentially expressed genes identified by RNA-seq. The Reactome bioinformatic tool was used for pathway analysis and identified a number of gene clusters known to promote myoblast fusion/differentiation.

To clarify the role of SATB2 in the regulation of genes important to myogenesis and the associated chromatin remodeling, we performed both ChIP- and RNA-seq analyses. Analysis of the ChIP-seq data indicated that SATB2 was bound throughout the proliferating muscle stem cell genome (Figure 3A) and was mainly positioned in the intergenic spaces (Figure 3C). That said, a significant number of SATB2 peaks were found within or adjacent to genes, including binding sites within promoters, introns, exons, and 3’ untranslated regions (Figure 3B for the binding site distance to the transcription start site (TSS) of the nearest gene, and Figure 3C for the binding site genomic context). An investigation into SATB2 binding motifs within these satellite cells revealed no conserved motifs on a genome-wide scale (Supplementary Table S2).

Given the canonical role of SATB proteins in regulating the chromatin structure and the proximity of SATB2 to the coding regions of many genes, a more thorough investigation into its impact on chromatin remodeling-associated gene expression changes was warranted. To this end, RNA-seq was performed on 24 h differentiated C_2_C_12_ cells that had been treated with either scrambled or SATB2 siRNA. Prior to our in-depth analyses of gene expression changes following SATB2 loss, we confirmed via RNA-seq that our siSatb2 treatment successfully downregulated *Satb2* mRNA (Supplementary Figure S3A). Using DESeq2 with an adjusted *p* value of 0.05 as a cut-off, our RNA-seq analyses revealed that the siRNA-mediated knockdown of SATB2 led to the reduced expression of 1510 genes and the increased expression of 1595 genes in 24 h differentiated myoblasts. Among the genes that were differentially expressed when SATB2 expression was altered were several prominent chromatin remodeling factors. These genes included *Setbp1*, *Hdac4/5/9/11*, and *Gata3* (Figure 3D). 

GO categorization of the genes that increased in expression following *Satb2* knockdown included various signaling genes and those involved in cell differentiation (Figure 3E); this included several chromatin modifying genes known to accelerate differentiation such as *Smarca4* [41,42,43], *Med21* [44], *Kat2b* [45], and *Bdnf* [46]. Alternatively, the GO categorization of those genes significantly downregulated upon SATB2 removal included genes involved in muscle development (Figure 3F). Several of these genes are known repressors of differentiation, such as *Hdac4* [47], *Hdac5* [47], *Ncoa1* [48], and *Mylk* [49], and may act to limit satellite cell activation and muscle growth. However, several of the suppressed genes are known mediators of differentiation, such as *Nfatc4*. Given that the removal of SATB2 accelerates myoblast differentiation, these changes may be a compensatory response to a myogenic program that is well underway.

To obtain a more granular view of SATB2′s role in regulating myogenic progression, we assessed the expression changes of genes that possessed a SATB2 binding site located −5 kb to + 1 kb from the gene TSS. GO categorization revealed that SATB2 was bound near the TSS of many genes involved in chromatin organization and modification (Supplementary Figure S3E). However, several genes associated with cell differentiation or proliferation were found to have SATB2 near its TSS (Supplementary Figure S3D), suggesting a limited role for SATB2 in managing gene expression within promoter boundaries. Finally, we undertook a complimentary unbiased bioinformatic assessment of gene expression in wildtype versus siSATB2 cells using GSEA (gene set enrichment analysis) coupled to the Reactome cluster profiling program. Similar to the GO categorization, GSEA identified an up regulation in a number of gene groups/pathways associated with chromatin/chromosome modification, including cell cycle checkpoint and chromatid cohesion categories, along with the down regulation of a subcluster of skeletal muscle contractile gene sets. Interestingly, unlike the GO categorization, GSEA revealed an upregulation in three gene categories known to separately enhance myoblast cell fusion/differentiation, including IL4 signaling, hyaluronan metabolism, and Rho GTPases/formin activation [50,51,52].

### 3.4. SATB2 Regulates Chromatin Organization around Genes Related to Myogenesis

**Figure 4:**
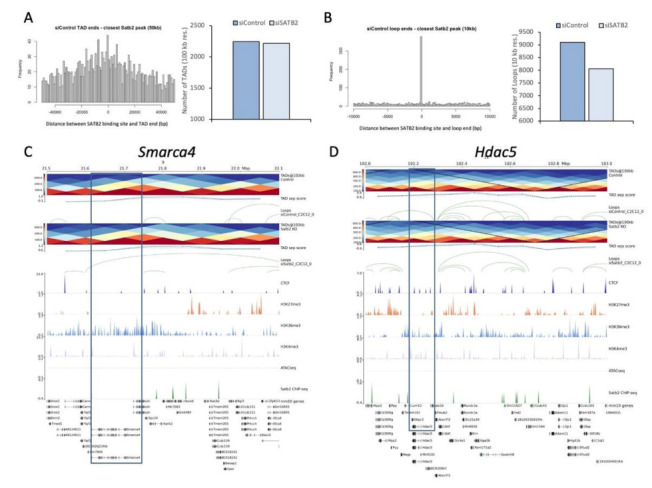
SATB2 is associated with chromatin loops and regulates loop dynamics in a subset of myogenesis-related genes. (**A**) Distribution of SATB2 binding sites with respect to TAD ends (50 kb resolution), as determined by Juicer v.1.6. The bar graph depicts the number of TADs detected at 100 kb resolution between siControl- and siSATB2-treated proliferating C2C12 samples. (**B**) Distribution of SATB2 binding sites with respect to loop ends (10 kb resolution), as determined by Juicer v.1.6. The bar graph depicts the number of loops detected at this resolution between siControl- and siSATB2-treated samples. (**C**) Chromatin structural landscape around the *Smarca4* locus. TADs and loops were assessed at 100 and 10 kb resolutions, respectively, using the tools described in (**A**,**B**). CTCF binding motifs were obtained from CTCF ChIP-seq data in mouse C2C12 cells from ENCODE (ENCSR000AIJ). The location of repressive (H3K27me3) and permissive (H3K4me3 and H3K36me3) histone marks were obtained by Asp et al. [35] from proliferating C2C12 myoblasts. The accession numbers for the associated ChIP-seq data are: H3K4me3 (GEO accession: GSM721292), H3K27me3 (GEO accession: GSM721294), and H3K36me3 (GEO accession: GSM721296). (**D**) Chromatin structural landscape around the *Hdac5* locus. The structural aspects depicted in the panel were obtained as described above.

SATB2 was initially characterized as a MAR protein; however, there are no genome-wide data examining the role of SATB2 as a chromatin organizing factor and how this protein may control genome structure. To address this issue directly, we performed a chromatin conformation capture using the Hi-C method and examined this genome structure data in relation to our SATB2 ChIP-seq data. This combined genome-wide analysis indicated that SATB2 binding sites were enriched near the ends of genetic loops and slightly enriched near the ends of topologically associated domains (TADs) (Figure 4A,B show the results for siControl samples). Interestingly, SATB2 binding sites are near loop ends but are not coincident with loop anchor points, as evidenced by the lack of direct overlap with CTCF binding motifs (data taken from ENCODE (ENCSR000AIJ) and the representative images shown in Figure 4C,D; Supplementary Figure S4B shows an absence of overlap for the SATB2 and CTCF binding sites), which are known markers of chromatin loop boundaries [53]. Given SATB2′s localization at the base of these three-dimensional genetic structures, one might predict that the loss of SATB2 in muscle cells would cause chromatin remodeling at various loci. Indeed, the loss of SATB2 in proliferating myoblasts caused a ~12% reduction in the number of loop structures and only a ~1% reduction in TADs (Figure 4A,B). 

The observation that SATB2 removal from muscle myoblasts affects chromatin loops and TADs suggests that SATB2 may manage gene expression through the direct modification of large-scale chromatin structures. In turn, the changes in chromatin organization may prepare muscle stem cells for differentiation by establishing the architecture needed for the efficient expression or repression of genes that manage the myogenic program. Indeed, the genetic locus surrounding the pro-differentiation factor, *Smarca4*, undergoes a transformation when SATB2 is lost, whereby a chromatin loop forms at the *Smarca4* site where one did not previously exist (Figure 4C). This loop structure is likely to facilitate *Smarca4* expression when cells are induced to differentiate. Conversely, for *Hdac5*, a chromatin loop containing the gene disappears following SATB2 ablation, which is concurrent with the repression of *Hdac5* expression during accelerated myogenesis (Figure 4D). This, however, may not be the only mechanism by which SATB2 contributes to gene expression changes, as several gene loci undergo subtle or limited local loop remodeling (Supplementary Figure S4). In these cases, SATB2 removal at more distal sites may have a broader effect on chromatin conformation that affects gene expression. An analysis of sub-TAD sized contact domains revealed alterations near several of the genes that lacked SATB2-derived chromatin loop remodeling. For instance, we observed changes in the number or width of contact domains present near *Ncoa1*, *Mylk*, and *Hdac4* (Supplementary Table S3). Interestingly, these types of changes were not observed in the genes that possessed local chromatin loop remodeling (i.e., *Smarca4*, *Hdac5*, and *Med21*).

The myogenic gene expression program has also been shown to be heavily influenced by epigenetic modifications, the most prominent of which is histone methylation, which acts to induce or repress expression within specific genes [54,55]. However, an investigation into the relationship between SATB2 binding sites and known muscle regulatory methylation sites such as the H3K27me3, H3K36me3, and H3K4me3 marks in proliferating myoblasts (taken from [35]) indicates that there is no significant correlation between histone methylation and SATB2 binding. 

### 3.5. In Vivo Depletion of SATB2 in Muscle Satellite Cells Decreases Muscle Fiber Area and the Number of Pax7-Expressing Satellite Cells 

**Figure 5:**
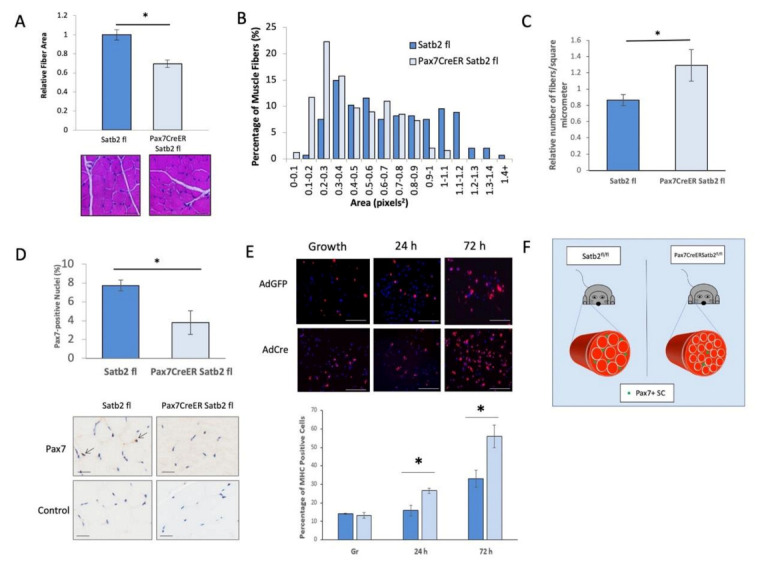
The in vivo reduction of SATB2 in muscle satellite cells decreases muscle fiber area and the number of Pax7-expressing satellite cells. (**A**) Tibialis anterior fiber areas decreased in *Pax7CreER/Satb2^fl/fl^* mice as compared to control (*Satb2^fl/fl^*) mice. ImageJ was used to measure the fiber areas of hematoxylin and eosin (H&E) stained tissue sections. Data are the means ± SEM, *n* = 3 independent determinations on separate tissue samples. H&E stained sections are representative of *n* = 3 independent determinations. Scale bar: 100 μm. (**B**) The distribution of muscle fiber sizes across 15 bins. Over 200 muscle fibers were measured for both control and *Pax7CreER/Satb2^fl/fl^* mice. (**C**) Bar graph indicating the significant increase in the relative number of muscle fibers per μm^2^. * indicates a significant difference between *Pax7CreER/Satb2^fl/fl^* and *Satb2^fl/fl^* mice, as determined by the Student’s *t*-test (*p* < 0.05). Data are the means ± SEM, *n* = 3 independent determinations on separate tissue samples. (**D**) The percentage of nuclei that were Pax7-positive in control (Satb2 fl) and Satb2-ablated (Pax7CreER Satb2 fl) mouse muscle. The panels below the bar graph are representative tissue sections stained with a Pax7 antibody (Pax7) or no antibody (Control); scale: 50 μm. Arrows indicate positively stained nuclei. Data are representative of *n* = 3 independent determinations on separate tissue samples. * indicates a significant difference between *Pax7CreER/Satb2^fl/fl^* and *Satb2^fl/fl^* mice, as determined by the Student’s *t*-test (*p* < 0.05). Data are means ± SEM for *n* = 3 independent determinations. (**E**) Immunofluorescent images (with accompanying bar graph) depicting an increase in myosin heavy chain (MHC) expression in Cre recombinase adenovirus (AdCre)-treated *SATB2^fl^* myoblasts as compared AdGFP-treated controls. Data are representative of three independent determinations on separate myoblast isolations. MHC (red); DAPI (blue). The histogram below the images represents the quantification of the number of cells that expressed MHC relative to the total number of cells counted. * indicates a statistically significant difference between AdGFP- and AdCre-treated samples using the Student’s *t*-test (*p* < 0.05). Data are means ± SEM for *n* = 3 independent determinations. Scale bars = 100 μm. (**F**) Graphical representation of the observed biology following in vivo SATB2 ablation (see Supplementary Figure S5A,B for confirmation of SATB2 knockdown) from muscle satellite cells in vivo. More specifically, with no change in muscle size (see Supplementary Figure S5C), SATB2 loss caused a significant reduction in muscle fiber size as well as the number of Pax7-expressing satellite cells.

To examine whether the loss of SATB2 would alter the muscle cell differentiation program in vivo, we conducted a preliminary analysis using a muscle progenitor cell-specific gene-targeted deletion of SATB2. Here, we generated the satellite cell-specific deletion of SATB2, under the control of tamoxifen induction, by crossing the *Satb2^fl^*^/*fl*^ mouse strain [56] with the *Pax7CreER* mouse strain [53] to generate *Pax7CreER*/*Satb2^fl^*^/*fl*^ mice. *Pax7CreER*/*Satb2^fl^*^/*fl*^ mice (and requisite controls, *Satb2^fl^*^/*fl*^ strain) were given tamoxifen at three weeks of age and monitored continuously (as per University of Ottawa Animal Care and Veterinary Service (ACVS) guidelines). The gross assessment of motor function and the physical state indicated that there were no substantial motor effects stemming from the depletion of SATB2. The disruption of SATB2 expression was confirmed via immunohistochemistry as well as western blots probing for SATB2 in myoblasts isolated from tamoxifen-treated mice (Supplementary Figure S5A,B). A quantitative assessment of muscle (extensor digitorum longus and TA) weights showed no significant differences between control and *Pax7CreER*/*Satb2^fl^*^/*fl*^ mice (Supplementary Figure S5C). However, a histologic/morphologic analysis revealed notable alterations in skeletal muscle structure between control and *Pax7CreER*/*Satb2^fl^*^/*fl*^ mice. For example, measurement of the fiber area within the TA from control and *Pax7CreER*/*Satb2^fl^*^/*fl*^ mice indicated that the removal of SATB2 from muscle satellite cells led to a significant decrease in fiber area concurrent with an increase in fiber number per µm^2^ as compared to the control strains (Figure 5A–C). Moreover, following tamoxifen treatment, the number of Pax7-expressing satellite cells within *Pax7CreER*/*Satb2^fl^*^/*fl*^ TA muscle decreased substantially when compared to the *Satb2^fl^*^/*fl*^ control muscle (Figure 5D). We also showed that primary myoblasts isolated from *Satb2^fl^*^/*fl*^ mice and treated with Cre adenovirus displayed enhanced differentiation kinetics (Figure 5E) that were similar to that observed in siSatb2-targeted C2C12 cells (Figure 1E). Indeed, these observations are consistent with the muscle hypotonia that often accompanies human SATB2 mutations [57].

## 4. Discussion

Chromatin remodeling plays a central role in stem cell differentiation as it facilitates the dramatic shift in gene expression profiles that accompanies the exit from the cell cycle and the commitment to a particular lineage [1,4,58,59,60,61,62]. These changes are dependent upon both transcription mediated change concurrent with a shift in the epigenetic landscape, which may allow or limit gene expression in the relevant regions of the genome. One mechanism that will influence lineage-dependent transcription and epigenetic change is the presence (or absence) of higher-order MAR proteins that mediate chromatin structure and thereby physical access to key genetic loci [10,63,64]. While changes to the chromatin architecture within skeletal muscle progenitor cells have been observed [5], the proteins that govern these changes are not well-known. Here, we identified SATB2 as a chromatin organizer that plays a key role in mediating the progression of myoblasts toward the myogenic differentiation program.

Interestingly, the reduction of SATB2 expression did not affect myoblast proliferation (Supplementary Figure S1B,C), indicating that SATB2 acts to block cells from prematurely entering into a terminally differentiated state rather than maintain a proliferation competent milieu. The reduced expression of SATB2 during early myogenesis appears to accelerate the differentiated phenotype, as evidenced by the siRNA-mediated knockdown of SATB2, which in turn hastens the expression of MHC (Supplementary Figure S1E), accelerates the formation of myotubes during early differentiation (Figure 1E), which is concurrent with the upregulation of factors that promote myoblast fusion (Figure 3G) [50,51,52]. This coincides with our supposition that SATB2 may sequester differentiation inductive genes and, once removed, primes myoblasts for to engage the myogenic program. While our observations indicate the clear anti-myogenic effect of SATB2 expression, others have shown that SATB2 may enhance muscle differentiation in transformed cell lines such as Sol8 cells [65]. However, this same study also reported a strong synergy between SATB2 and BMP-induced transcription activity, which is known to be robustly anti-myogenic, suggesting in fact that SATB2 does antagonize differentiation akin to our observations reported here.

Collectively, our observations suggest that the loss of SATB2 is coincident to the progress of the differentiation program. Therefore, the mechanism by which the cell manages the repression of SATB2, and whether this occurs through transcriptional or post-translational mechanisms, is of paramount interest. Our identification of a major C-terminal fragment of SATB2, which becomes more prominent during muscle cell differentiation, suggests that regulated proteolysis may target the SATB2 protein and that this may be a critical step in managing cell differentiation (Figure 1A and Figure S2D). Indeed, prior studies from our laboratory have shown that caspase 3 plays a prominent role in myogenesis, targeting and cleaving a number of proteins to engage the differentiation program [22,24,36,37]. In addition, evidence from apoptotic nuclei suggests that nuclear structural proteins are targeted by caspase proteases as part of a chromatin dissolution mechanism [19,39,40]. 

Despite the prominent role of caspase 3 in the differentiation process, we have noted that SATB2 is cleaved exclusively by the effector caspase, caspase 7 (Figure 2C). Suppression of this protease in vitro led to the sustained expression of SATB2 (Figure 2D,E), with a concomitant reduction in myogenesis (Figure 2G). Cytosolic caspase 7 activity has been suggested to promote odontogenesis [66,67], yet nuclear caspase 7 disposition is considered to be an exclusive hallmark of apoptosis [67,68]. For example, remodeling the chromatin micro-environment through targeted protein cleavage events is considered to be a conserved feature of apoptosis, as exemplified by the effector caspase cleavage of scaffold attachment factor b1 [69], lamina-associated polypeptide 2α [70], and SMARCA2 and SMARCA4 [40]. Nevertheless, our observations support a novel model whereby an effector caspase, caspase 7, targets a protein substrate (SATB2) in order to prime the nuclear matrix to engage differentiation, independent of cell death. Presumably, during muscle cell differentiation, the activation of caspase 7 is mediated by the same pathway that leads to the induction of caspase 3 via the engagement of the mitochondrial intrinsic cell death pathway [23]. The pattern of caspase 7 activation is remarkably similar to caspase 3 in differentiating myoblasts (Figure 2F and Figure S2E), which does support a common signaling origin. What is more speculative is whether a level of integration may exist between caspase 3 and caspase 7 activity, where the targeting of their respective substrates is coordinated to drive the same biologic alteration. 

Central to SATB2′s role in controlling gene accessibility and expression is its impact on the chromatin structure during myoblast differentiation. Our ChIP-seq analyses indicate that SATB2 is bound throughout the mouse myoblast genome (Figure 3A), mainly in the intergenic spaces, but also near or within the coding regions of many genes (Figure 3C and integrated genomics viewer/IGV display for SATB2 targets, Supplementary Figure S5). The widespread distribution of SATB2 is consistent with the localization generally observed for matrix attachment region proteins [71]. However, the binding sites of SATB2 are positioned near but independent from chromatin loop anchor points (Figure 4B and Figure S4B), indicating a hitherto unappreciated role in maintaining the three-dimensional chromatin architecture. This observation is somewhat in contrast to recent work, where neuronal SATB2 was found to be associated with the nuclear lamina via LEMD2, and the ablation of either protein resulted in substantial changes to nuclear organization and gene regulation [17]. Accordingly, our study adds critical insight into the entities that govern chromatin organization and cell function, suggesting that in a myogenic cell, SATB2 actively modifies chromatin architecture by establishing gene expression boundaries through the formation of loops and TAD-like domains. Whether SATB2-dependent loops and TADs follow a specific subnuclear distribution, i.e., preferential location at the nuclear edge, and whether this occurs in other cell lineages will require further investigation.

The loss of myoblast SATB2 affects the genetic landscape in several ways. Most directly, SATB2 ablation led to changes in three-dimensional chromatin folding, as evidenced by the reduction in chromatin loops following its suppression (Figure 4B). SATB2′s primary association with loop ends, as opposed to TAD ends, adheres to the original structural role described for the SATB family of proteins [72,73,74,75]. For example, SATB1 has been shown to mediate the formation of chromatin in helper T cells and is necessary for the expression of key cytokines following T cell activation [74]. In addition to affecting gene accessibility through chromatin loop remodeling, SATB2 loss appears to affect the expression of several important chromatin modifiers such as *Hdac4/5/7/9* (Figure 3D), which themselves can further alter the genetic architecture during myogenesis and modify the activity of key transcription factors such as the Mef2 family. These effects likely form the foundation for many of the gene expression changes observed in this study and may serve to open/close genetic locales that would support the progression of the myogenic program. A prime example of this would be for *Smarca4*, which typically supports cell differentiation [41,42,43]. When SATB2 is suppressed, *Smarca4* becomes part of a new loop structure, which may facilitate its expression during myogenesis (Figure 4C). Conversely, *Hdac5* exits a SATB2-mediated loop structure when the latter is ablated, which may serve to help repress its expression and aid in the progression of the myogenic program (Figure 4D). 

While changes in local chromatin folding may be the primary driver of SATB2-associated gene expression changes, our data suggest that some changes may occur by means of an independent mechanism, potentially through changes in sub-TAD contact domains or changes in the DNA looping that influences distal target genes [76]. For instance, the local chromatin loops around *Ncoa1* and *Hdac4* appear virtually unaltered between siControl and siSATB2 treatments (Supplementary Figure S4C). However, the sub-TAD domains at these loci changed following SATB2 suppression, with either domains being lost or altered in size (Supplementary Table S3). These sub-TAD domains can be critical regulators of gene expression and may lie within a larger TAD superstructure that remains unaltered [77,78]. Moreover, evidence exists that changes in sub-megabase contact domains may be critical for the fidelity of gene expression during embryonic stem cell and neural progenitor cell fate determination [79]. Interestingly, those genes (*Smarca4*, *Hdac5*, and *Med21*) that possessed changes in local loop structures did not possess contact domains at the resolutions tested, suggesting that these modes of regulation may be mutually exclusive. Taken together, SATB2′s impact on genome loop structures and sub-TAD level contact domains appear to hamper myoblast differentiation, necessitating SATB2 removal for the induction of chromatin reorganization that facilitates the myogenic differentiation program. 

In addition to alterations in local chromatin environments, others have noted that skeletal muscle differentiation coincides with macro nuclear alterations, where transcriptions factors and chromatin remodeling proteins, working in concert, reduce inter-chromosomal contacts at critical differentiation-specific loci [33]. We have noted that the loss of SATB2 expression is concurrent with a global reduction in the number of inter-chromosomal links (5.25 ± 0.22 vs. 5.90 ± 0.01%, *p* = 0.04, see Supplementary Table S5), suggesting that SATB2 may similarly modify this macro nuclear structure, perhaps reducing inter-chromosomal links that repress the differentiation program. However, an accurate identification of such SATB2 sensitive loci will require alternative 3C experiments that provide greater sequence depth compared to our current Hi-C approach [80,81].

In closing, our data do not favor a model whereby SATB2 binds to any traditional chromatin anchor. Rather, our data suggest that SATB2 binds within the looped chromatin, proximal to CTCF and its cohesin-linked protein complexes, which together anchor the loop structure (as defined in [82]). Furthermore, the very modest association of SATB2 at anchor points implies a loop-oriented binding event rather than a direct physical association with CTCF per se (we also see SATB2 bind within a cohort of subTAD domains, which again reinforces the loop-binding propensity of this protein). Intra-chromatin loop protein binding (and the regulation of gene expression) has not been reported in the literature previously; however, our data are consistent with this novel and exciting hypothesis. Indeed, a very recent publication has shown that the Jpx RNA can bind directly within a chromatin loop, modify its structure, separately impair CTCF binding kinetics, which all together leads to altered gene expression [83]. This aforementioned study together with our observations suggest a new and unappreciated mechanism for controlling the local chromatin structure, affecting gene expression, and influencing cell fate decisions.

## Data Availability

Mass spectrometry data are available in the MassIVE repository under the identifier MSV000085254. Genomics (ChIP-seq, RNA-seq, and Hi-C) data are available using GEO accession GSE185437.

## Supplementary Materials

The following supporting information can be downloaded at: https://www.mdpi.com/article/10.3390/cells11060966/s1, Figure S1: SATB2 expression represses myoblast differentiation, Figure S2: Caspase 7 cleaves SATB2 during early skeletal muscle differentiation and is necessary for myogenesis, Figure S3: Quality control and validation data related to ChIP- and RNA-seq experiments, Figure S4: Validation data for SATB2 genomic analysis, Figure S5: Integrative Genomics Viewer (IGV) tracks depicting select genes (blue) related to proliferation/differentiation and the SATB2 binding sites within that area of the myoblast genome (grey), Figure S6: Validation of SATB2 loss following tamoxifen treatment of *Satb2^fl/fl^* and *Pax7CreER/Satb2^fl/fl^* mice, Table S1: Mass spectrometry analysis of the major cleavage fragment of SATB2, Table S2: Top 10 enriched DNA sequence motifs within the full set of SATB2 ChIP peaks, Table S3: Sub-TAD contact domains identified near *Ncoa1*, *Hdac4*, and *Mylk* genes for siControl- and siSATB2-treated proliferating C2C12 cells, Table S4: ChIP-seq statistics for proliferating C2C12 cells, Table S5: HiC replicate statistics for siControl- and siSATB2-treated proliferating C2C12 cells
