# Supplementary material for: Chromatin Reorganization during Myoblast Differentiation Involves the Caspase-Dependent Removal of SATB2
Source: Cells. 2022 Mar 11;11(6):966. doi: 10.3390/cells11060966 (PMC8946544; doi:10.3390/cells11060966)
Supplement: Supplementary file 1 [file cells-11-00966-s001.zip › cells-1538621-supplementary.pdf]

Supplemental Information

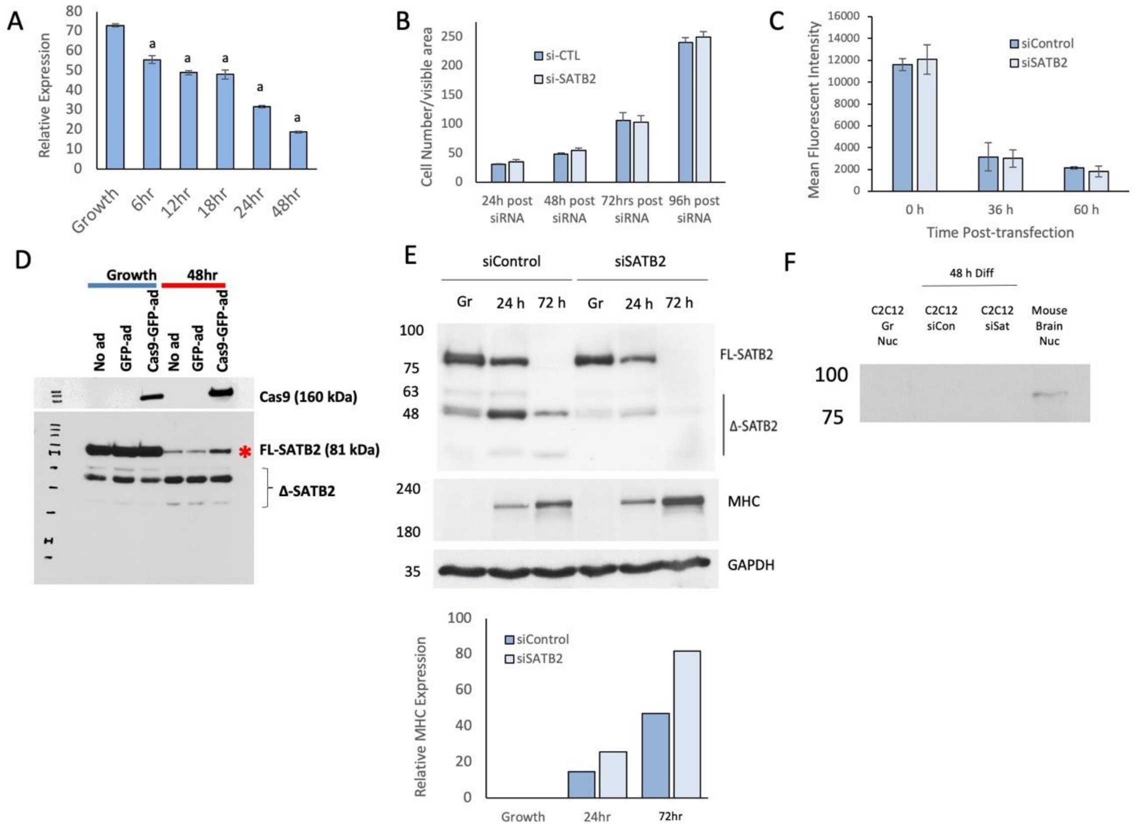

Supplementary Figure S1. SATB2 expression represses myoblast differentiation. (A) Quantification of SATB2 expression in C2C12 cells during differentiation depicted in the western blot from Figure 1A. a - indicates a significant difference in SATB2 expression at the differentiation timepoint as compared to the growth condition using the Student's *t*-test ($p < 0.05$). (B) siRNA-mediated depletion of SATB2 does not affect C2C12 proliferation and survival. C2C12 cells were counted using bright field microscopic images under low magnification. Each individual cell in a field was marked and counted using ImageJ cell counter software. Data are means ± SEM for n = 3 independent determinations. No statistically significant difference in cell number was observed between siControl and siSATB2 treatments at each time point. (C) CFSE fluorescence was assessed by an ArrayScan imaging system in proliferating C2C12 cells that had been treated with control or SATB2 siRNA. CFSE fluorescence becomes reduced as cells divide and differences in proliferation ability would be detected through perturbations in mean fluorescent intensity from the treated cells. No significant difference was observed in the average mean-intensity of the cells' fluorescence up to 60 h after siRNA treatment (when 100% confluence was reached). Data are means ± SEM for n = 3 independent determinations. (D) SATB2 expression following adenoviral Cas9 transfection into C2C12 myoblasts. SATB2 expression increased following Cas9-GFP-ad transfection as compared to no transfection and GFP-ad transfection controls (48 h differentiated C2C12 cells; n = 2). * indicates the location of full-length SATB2. Cas9 expression was confirmed in the Cas9-GFP-ad cells

and is shown in a panel above the main blot. (E) Western blot showing an increase in myosin heavy chain (MHC) protein expression and decrease in SATB2 expression in siSATB2-treated cells as compared to siControl-treated cells during differentiation time points. Image is representative of n = 2 independent determinations. GAPDH was used as a loading control. Bar graph to the right of the western blot represents the quantification MHC shown in the blot. (F) SATB1 was not found in nuclear extracts from growing or 48 h differentiated C2C12 cells and was only found in the mouse brain sample (positive control).

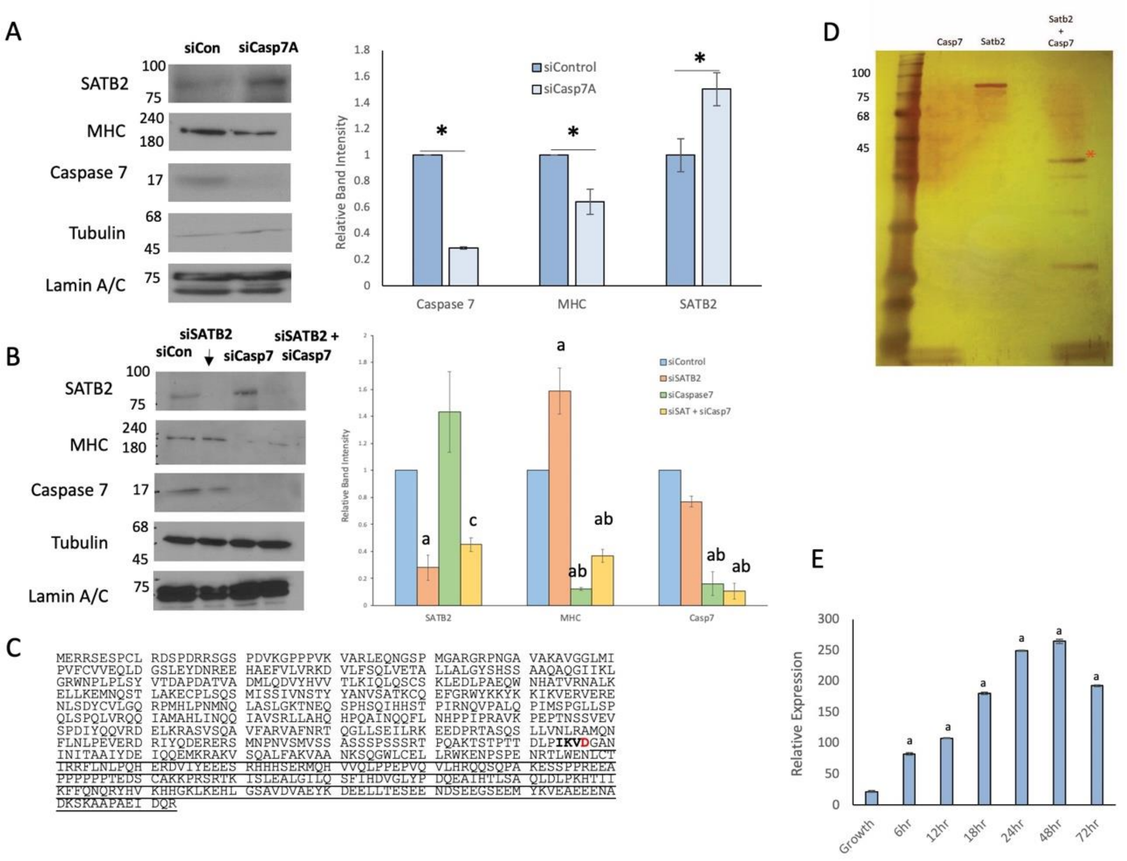

Supplementary Figure S2. Caspase 7 cleaves SATB2 during early skeletal muscle differentiation and is necessary for myogenesis. (A) Decreased expression of caspase 7 in 48 h differentiated C2C12 cells by an alternative siRNA (siCasp7A) from the siRNA reported in Figure 2. * indicates a significant difference in expression when siSATB2 is compared to siControl using the Student's *t*-test. Data are means ± SEM for n = 3 independent determinations. (B) Knockdown of caspase 7 and SATB2 concomitantly in 48 h differentiated C2C12 cells rescues MHC expression. a – indicates a significant difference between siSATB2 and siControl samples; b – indicates a significant difference between siCaspase7 and siControl samples; c – indicates a significant difference between siSATB2 + siCaspase 7 and siCaspase7 samples. Statistical differences were assessed by a one-way ANOVA with a post-hoc Tukey test ($p < 0.05$). Data are means ± SEM for n = 3 independent determinations. (C) The amino acid sequence of *Mus musculus* SATB2, with the C-terminal cleavage fragment underlined and the caspase cleavage site bolded. The red 'D' indicates the aspartic acid residue where cleavage occurs. (D) Silver stained gel of an in vitro caspase cleavage assay involving recombinant SATB2 and active caspase 7. * indicates the major cleavage fragment that was analyzed by mass spectrometry and included the D477 cleavage site. (E) Quantification of the relative active caspase 7 expression during C2C12 myoblast differentiation (depicted in Figure 2F). a – indicates a significant difference in active caspase 7 expression during C2C12 differentiation as compared to the growth condition using the Student's *t*-test ($p < 0.05$).

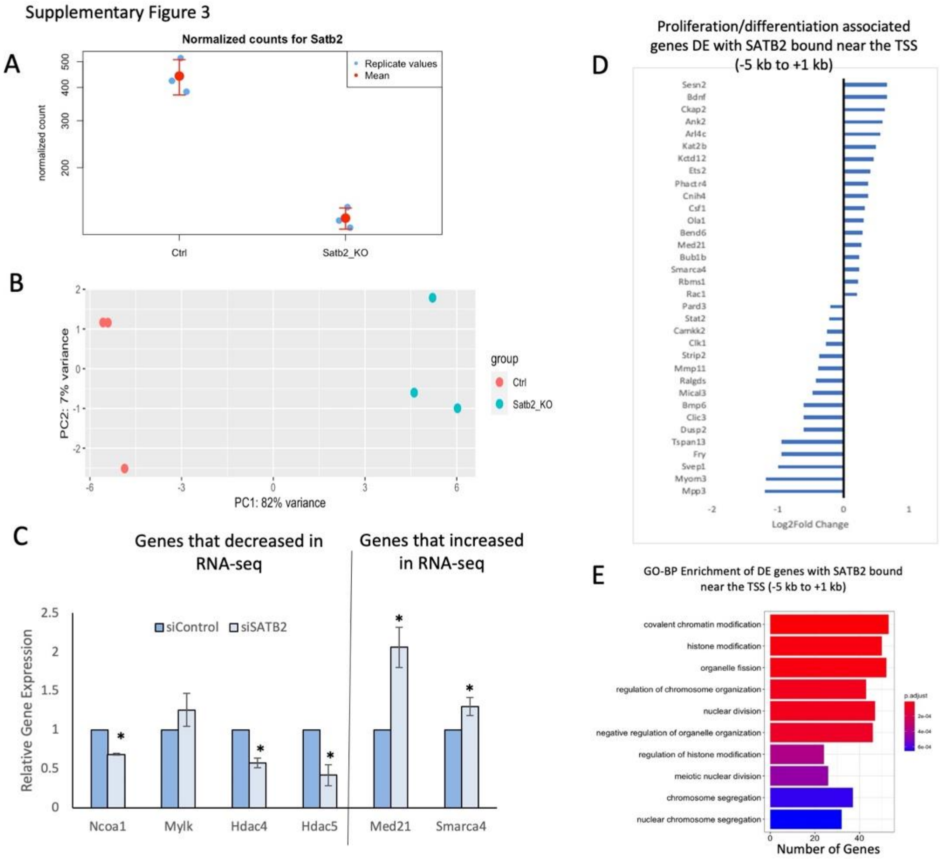

Supplementary Figure S3. Quality control and validation data related to ChIP- and RNA-seq experiments. (A) Validation of SATB2 downregulation following siSATB2 treatment via RNA-seq. (B) PCA plot assessing the variance in the RNA-seq replicates. The majority of the variance is between siControl and siSATB2 treatments and replicates are well correlated. (C) RT-PCR validation of gene expression changes observed in our RNA-seq data. Histogram includes several genes that decreased in expression following *Satb2* suppression (*Ncoa1*, *Mylk*, *Hdac4*, and *Hdac5*) as well as two genes that increased in expression under the same conditions (*Smarca4* and *Med21*). * indicates a significantly different level of expression for that gene under the siSATB2 condition as compared to the control ($p < 0.05$; Student's *t*-test). Data are means ± SEM for $n = 3$ independent determinations. (D) Selection of proliferation/ differentiation associated genes that had SATB2 bound near their TSS (-5 kb to +1 kb) and were differentially expressed when *Satb2* expression was inhibited. (E) The top GO-BP categories for the differentially expressed genes that possessed SATB2 bound near the TSS (-5 kb to +1 kb). Analysis in E was provided by ClusterProfiler.

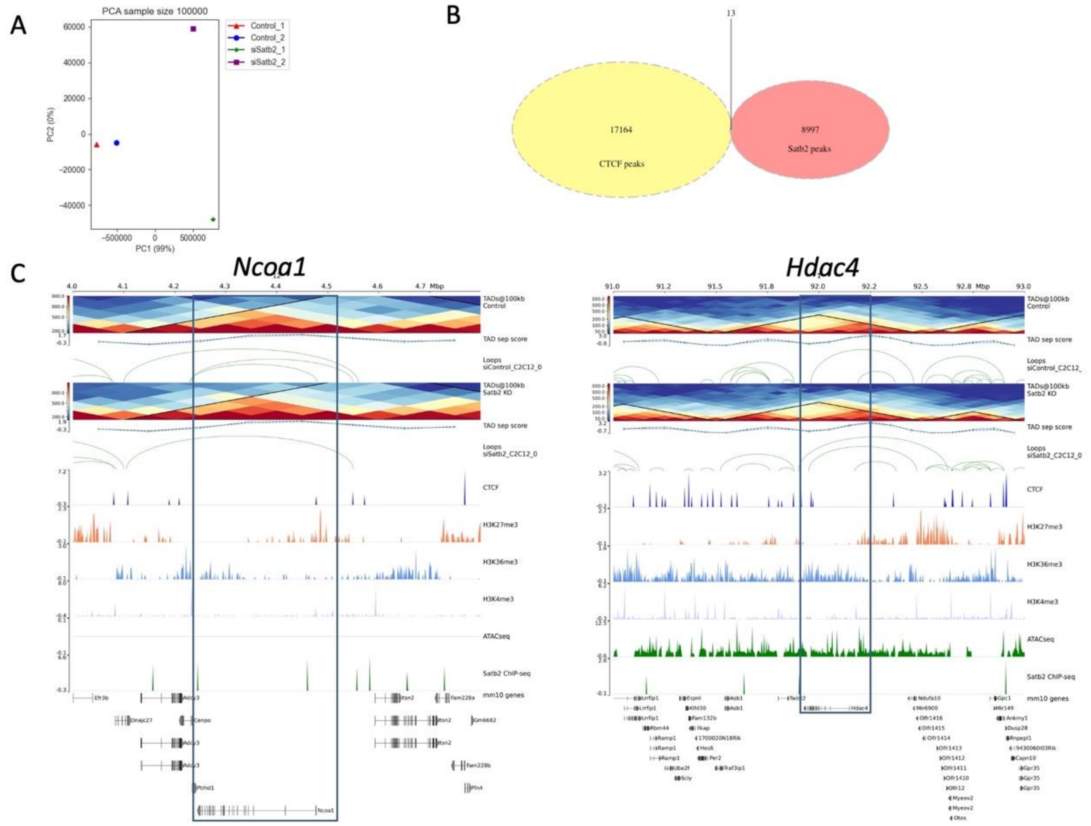

Supplementary Figure S4. Validation data for SATB2 genomic analysis. (A) Intra-treatment replicates were more closely correlated than inter-treatment replicates with respect to our HiC data. (B) Venn diagram of CTCF binding sites and SATB2 binding sites within the proliferating C2C12 myoblast genome. CTCF binding sites were generally independent of SATB2 binding sites. (C) TAD and loop structures around *Ncoa1* and *Hdac4* remain virtually unchanged when SATB2 is removed from proliferating C2C12 cells as determined by our Hi-C analyses. This corresponds with a lack of SATB2 binding sites at these loci, suggesting an alternate mechanism by which these genes are regulated. Included in these panels are CTCF binding sites from proliferating myoblasts (taken from ENCODE (ENCSR000AIJ)) as well as local histone methylation markers (H3K27me3, H3K36me3, and H3K4me3; taken from Asp et al., 2011). See the Materials and Methods for details regarding CTCF and methylation data sets.

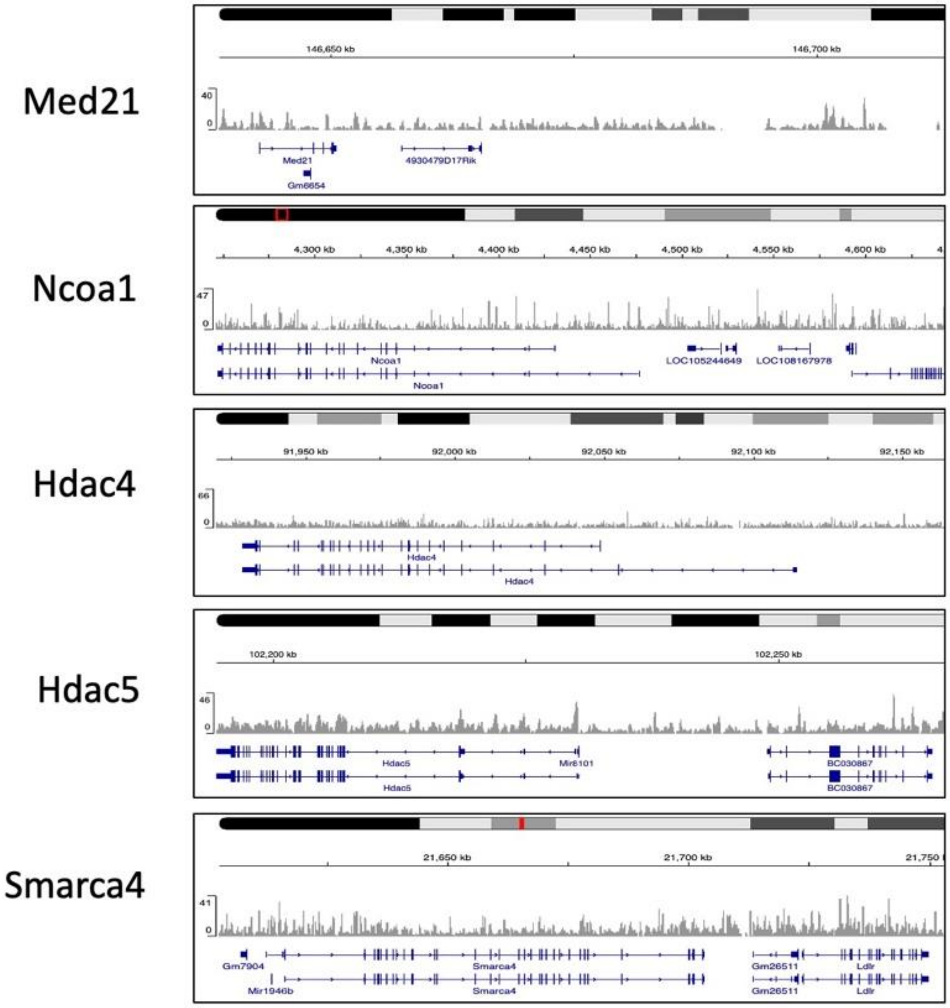

Supplementary Figure S5. Integrative Genomics Viewer (IGV) tracks depicting select genes (blue) related to proliferation/differentiation and the SATB2 binding sites within that area of the myoblast genome (grey).

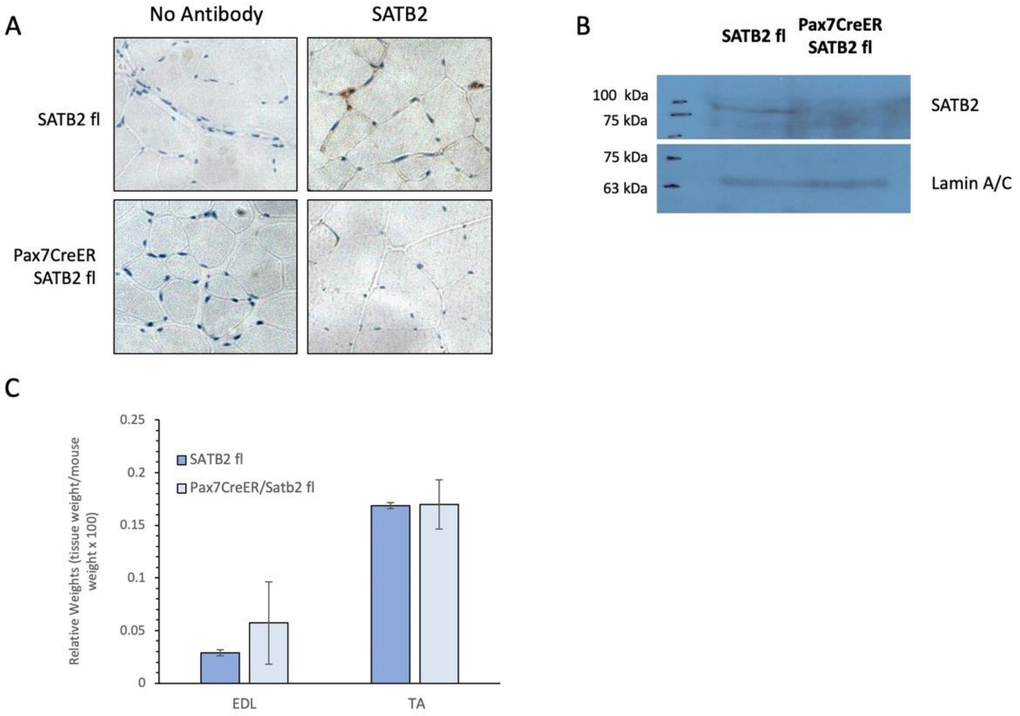

Supplementary Figure S6. Validation of SATB2 loss following tamoxifen treatment of *Satb2*$^{fl/fl}$ and *Pax7CreER/Satb2*$^{fl/fl}$ mice. (A) Immunohistochemical staining of SATB2 in the tibialis anterior (TA) following Cre-induced loss of *Satb2* from muscle stem cells. (B) Western blot for SATB2 using myoblasts derived from tamoxifen-treated *Satb2*$^{fl/fl}$ and *Pax7CreER/Satb2*$^{fl/fl}$ mice. Lamin A/C expression was used as a loading control. (C) Weights for extensor digitorum longus (EDL) and TA muscles from tamoxifen-treated *Satb2*$^{fl/fl}$ and *Pax7CreER/Satb2*$^{fl/fl}$ mice.

Supplementary Table S1. Mass spectrometry analysis of the major cleavage fragment of SATB2, identified with an * in Supplementary Figure 2D.

Start-End	Observed	Mr(expt)	Mr(calc)	ppm	M	Score	Expect	Rank	U	Peptide
478-496	1069.5300	2137.0455	2137.0361	4.41	0	77	1.8e-06	1	U	D.GANINITAAIYDEIQQEMK.R
478-496	1069.5302	2137.0458	2137.0361	4.52	0	41	0.037	1	U	D.GANINITAAIYDEIQQEMK.R
478-497	760-0575	2277.1507	2277.1507	3.68	1	51	0.0027	1	U	D.GANINITAAIYDEIQQEMKR.A
478-497	1139.5853	2277.1561	2277.1423	6.07	1	48	0.00016	1	U	D.GANINITAAIYDEIQQEMKR.A
478-497	765.3890	2293.1451	2293.1372	3.45	1	18	0.58	1	U	D.GANINITAAIYDEIQQEMKR.A
478-497	765.3898	2293.1475	2293.1372	4.49	1	15	0.59	1	U	D.GANINITAAIYDEIQQEMKR.A
482-497	647.6520	1939.9341	1939.9196	7.45	1	7	1.6	1	U	I.NITAAIYDEIQQEMKR.A + 2
498-507	531.8209	1061.6273	1061.6233	3.76	1	7	0.23	1	U	R.AKVSQALFAK.V

Supplementary Table S2. Top 10 enriched DNA sequence motifs within the full set of SATB2 ChIP peaks.

Rank	Motif	P-value	log P-pvalue	% of Targets	% of Background	STD(Bg STD)
1	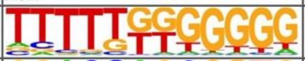	1e-19	-4.471e+01	2.49%	0.24%	70.8bp (63.6bp)
2	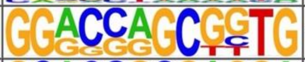	1e-12	-2.822e+01	1.20%	0.07%	53.0bp (56.5bp)
3 *	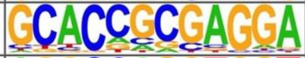	1e-9	-2.102e+01	2.58%	0.66%	56.0bp (56.2bp)
4 *	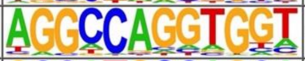	1e-7	-1.811e+01	2.58%	0.76%	57.6bp (63.5bp)
5 *	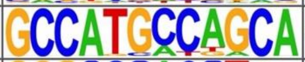	1e-7	-1.801e+01	0.69%	0.04%	49.0bp (41.4bp)
6 *	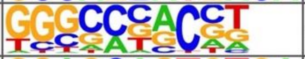	1e-7	-1.627e+01	1.63%	0.36%	54.3bp (60.6bp)
7 *	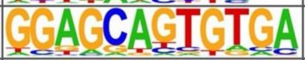	1e-7	-1.626e+01	3.61%	1.42%	57.6bp (66.0bp)
8 *	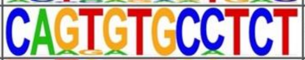	1e-6	-1.540e+01	0.43%	0.01%	52.4bp (58.8bp)
9 *	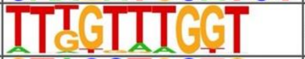	1e-6	-1.500e+01	2.49%	0.83%	71.5bp (66.2bp)
10 *	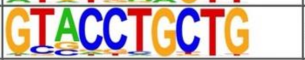	1e-5	-1.360e+01	3.35%	1.41%	54.2bp (65.5bp)

Analysis was conducted by HOMER v.4.11.1 using the default parameters. The red star indicates possible false positives within the data set.

Supplementary Table S3. Sub-TAD contact domains identified near *Ncoa1*, *Hdac4*, and *Mylk* genes for siControl- and siSATB2-treated proliferating C2C12 cells.

	Start	End	Width (bases)
**Ncoa1**			
siControl	4220001	4290000	70000
siControl	4290001	4480000	190000
siSatb2	4220001	4470000	250000
**Hdac4**			
siControl	91910001	91970000	60000
siControl	91970001	92180000	210000
siControl	92180001	92360000	180000
siSatb2	91910001	91960000	50000
siSatb2	91960001	92180000	220000
siSatb2	92180001	92380000	200000
**Mylk**			
siControl	34730001	34900000	170000
siControl	34900001	35310000	410000
siSatb2	34710001	34890000	180000
siSatb2	34890001	35050000	160000

Contact domains were determined from chromatin conformation capture, Hi-C, experiments using HiCexplorer v.3.6 at a resolution of 10 kb. The width column represents the size of the identified contact domain in bases.

Supplementary Table S4. ChIP-seq statistics for proliferating C2C12 cells.

Name	Total Reads	Mapped	Unique	No Duplicates	Duplicates
Growth C2C12 IgG control	82731365	80448079	50208240	33151090	17057150
Growth C2C12 SATB2 ChIP-seq	83088073	79335624	41712723	34014417	7698306

Supplementary Table S5. HiC replicate statistics for siControl- and siSATB2-treated proliferating C2C12 cells.

Attribute	siControl_C2C12	siControl_C2C12_1	siControl_C2C12_2	siSatb2_C2C12	siSatb2_C2C12_1	siSatb2_C2C12_2
**Sequenced Read Pairs**	880,135,554	442,256,173	437,879,381	926,871,724	460,599,259	466,272,465
**Normal Paired**	400,590,761 (45.51%)	206,734,525 (46.75%)	193,856,236 (44.27%)	390,338,648 (42.11%)	183,774,360 (39.90%)	206,564,288 (44.30%)
**Chimeric Paired**	382,620,864 (43.47%)	188,686,147 (42.66%)	193,934,717 (44.29%)	436,265,480 (47.07%)	223,753,096 (48.58%)	212,512,384 (45.58%)
**Chimeric Ambiguous**	81,719,897 (9.28%)	41,260,676 (9.33%)	40,459,221 (9.24%)	93,589,981 (10.10%)	49,639,820 (10.78%)	43,950,161 (9.43%)
**Unmapped**	15,204,032 (1.73%)	5,574,825 (1.26%)	9,629,207 (2.20%)	6,677,615 (0.72%)	3,431,983 (0.75%)	3,245,632 (0.70%)
**Ligation Motif Present**	0 (0.00%)	0 (0.00%)	0 (0.00%)	0 (0.00%)	0 (0.00%)	0 (0.00%)
**Alignable (Normal+Chimeric Paired)**	783,211,625 (88.99%)	395,420,672 (89.41%)	387,790,953 (88.56%)	826,604,128 (89.18%)	407,527,456 (88.48%)	419,076,672 (89.88%)
**Unique Reads**	654,781,728 (74.40%)	331,307,850 (74.91%)	326,221,815 (74.50%)	599,978,186 (64.73%)	301,521,148 (65.46%)	300,437,357 (64.43%)
**PCR Duplicates**	121,393,914 (13.79%)	60,732,579 (13.73%)	57,886,721 (13.22%)	217,409,584 (23.46%)	101,370,299 (22.01%)	114,032,786 (24.46%)
**Optical Duplicates**	7,035,983 (0.80%)	3,380,243 (0.76%)	3,682,417 (0.84%)	9,216,358 (0.99%)	4,636,009 (1.01%)	4,606,529 (0.99%)
**Library Complexity Estimate**	2,215,280,797	1,130,982,549	1,142,840,237	1,249,919,616	659,795,813	607,592,382
**Intra-fragment Reads**	0 (0.00% / 0.00%)	0 (0.00% / 0.00%)	0 (0.00% / 0.00%)	0 (0.00% / 0.00%)	0 (0.00% / 0.00%)	0 (0.00% / 0.00%)
**Below MAPQ Threshold**	119,246,539 (13.55% / 18.21%)	60,014,678 (13.57% / 18.11%)	59,911,136 (13.68% / 18.37%)	116,516,900 (12.57% / 19.42%)	58,757,276 (12.76% / 19.49%)	58,269,092 (12.50% / 19.39%)

**Hi-C Contacts**	535,535,189 (60.85% / 81.79%)	271,293,172 (61.34% / 81.89%)	266,310,679 (60.82% / 81.63%)	483,461,286 (52.16% / 80.58%)	242,763,872 (52.71% / 80.51%)	242,168,265 (51.94% / 80.61%)
**Ligation Motif Present**	0 (0.00% / 0.00%)	0 (0.00% / 0.00%)	0 (0.00% / 0.00%)	0 (0.00% / 0.00%)	0 (0.00% / 0.00%)	0 (0.00% / 0.00%)
**3' Bias (Long Range)**	50% - 50%	50% - 50%	50% - 50%	50% - 50%	50% - 50%	50% - 50%
**Pair Type %(L-I-O-R)**	25% - 25% - 25% - 25%	25% - 25% - 25% - 25%	25% - 25% - 25% - 25%	25% - 25% - 25% - 25%	25% - 25% - 25% - 25%	25% - 25% - 25% - 25%
**Inter-chromosomal**	51,965,608 (5.90% / 7.94%)	26,059,447 (5.89% / 7.87%)	25,908,307 (5.92% / 7.94%)	48,648,406 (5.25% / 8.11%)	26,000,675 (5.64% / 8.62%)	22,649,651 (4.86% / 7.54%)
**Intra-chromosomal**	483,569,581 (54.94 % / 73.85%)	245,233,725 (55.45 % / 74.02%)	240,402,372 (54.90 % / 73.69%)	434,812,880 (46.91 % / 72.47%)	216,763,197 (47.06 % / 71.89%)	219,518,614 (47.08 % / 73.07%)
**Short Range (<20Kb)**	235,297,149 (26.73 % / 35.94%)	121,584,695 (27.49 % / 36.70%)	115,776,732 (26.44 % / 35.49%)	190,895,350 (20.60 % / 31.82%)	87,671,941 (19.03% / 29.08%)	104,690,335 (22.45 % / 34.85%)
**Long Range (>20Kb)**	248,202,195 (28.20 % / 37.91%)	123,615,612 (27.95 % / 37.31%)	124,588,227 (28.45 % / 38.19%)	243,860,280 (26.31 % / 40.64%)	129,061,002 (28.02 % / 42.80%)	114,800,815 (24.62 % / 38.21%)
